# Detecting task-relevant spatiotemporal modules and their relation to motor adaptation

**DOI:** 10.1371/journal.pone.0275820

**Published:** 2022-10-07

**Authors:** Masato Inoue, Daisuke Furuki, Ken Takiyama

**Affiliations:** Department of Electrical Engineering and Computer Science, Tokyo University of Agriculture and Technology, Koganei, Tokyo, Japan; Tokai University, JAPAN

## Abstract

How does the central nervous system (CNS) control our bodies, including hundreds of degrees of freedom (DoFs)? A hypothesis to reduce the number of DoFs posits that the CNS controls groups of joints or muscles (i.e., modules) rather than each joint or muscle independently. Another hypothesis posits that the CNS primarily controls motion components relevant to task achievements (i.e., task-relevant components). Although the two hypotheses are examined intensively, the relationship between the two concepts remains unknown, e.g., unimportant modules may possess task-relevant information. Here, we propose a framework of task-relevant modules, i.e., modules relevant to task achievements, while combining the two concepts mentioned above in a data-driven manner. To examine the possible role of the task-relevant modules, we examined the modulation of the task-relevant modules in a motor adaptation paradigm in which trial-to-trial modifications of motor output are observable. The task-relevant modules, rather than conventional modules, showed adaptation-dependent modulations, indicating the relevance of task-relevant modules to trial-to-trial updates of motor output. Our method provides insight into motor control and adaptation via an integrated framework of modules and task-relevant components.

## Introduction

How our body is controlled is a fundamental question in motor neuroscience, biomechanics, and related areas. One difficulty in solving this question is the significant number of degrees of freedom (DoFs) inherent in our body, such as the large number of muscles and joints. The number of DoFs is more than necessary to achieve planned movements in large cases, causing a redundancy problem [1]. The redundancy problem refers to the fact that a large number of combinations of muscle activities and joint angles result in a unique movement pattern. Solving the redundancy problem in any way is indispensable for achieving planned movements.

A possible solution to the redundancy problem is to control groups of joints and muscles rather than an individual joint or muscle [1]. Such grouping entails a decrease in the number of DoFs. After the reduction in DoFs while grouping joints or muscles, time-varying motor commands are appropriately determined in each group. This framework is supported in diverse motor repertoires [2–7]. In locomotion, three joint angles were decomposed into two groups [5]. In walking, five groups of muscles were enough to reconstruct the muscle activities of 12–16 muscles [3]. In this framework, motion is assumed to be determined by the following two steps: 1) groups of muscles or joints are determined, and 2) the time-varying motor command is sent to the groups, resulting in the determination of muscle activities or joint angles. Due to this hierarchical structure of spatial features (i.e., step 1 mentioned above) and temporal features (i.e., step 2 mentioned above), the groups of muscles and joints are referred to as spatial modules [2–4]. Similarly, the time-varying motor commands sent to the spatial modules are referred to as temporal modules. In switching from walking to running, the peak timings of the temporal modules show modulations [6, 7] in addition to the modulation of the spatial modules [7]. In summary, constructing spatial and temporal modules and modulating the modules are possible candidate methods to overcome the redundancy problem and achieve planned movements.

Another possible solution to the redundancy problem is to decompose time-varying and multidimensional motion elements into task-relevant and task-irrelevant components [1]. Task-relevant components must be lower-dimensional compared to the original motion dimension, resulting in a reduction in the number of DoFs. Thus, it is possible to overcome the redundancy problem by primarily controlling task-relevant components. Notably, to define task-relevant and task-irrelevant components, how motion is relevant to task achievements should be clarified (e.g., in throwing a ball, the position and squared velocity of the ball at the release timing determine its maximum height, and in arm-reaching movements, the distance between the hand position and targeted position determines task achievement). After finding the relationship, it is possible to decompose time-varying and multidimensional motion elements into task-relevant components with low dimensions and task-irrelevant components with high dimensions. As evidence of this concept, the variability in the task-relevant dimension is lower than that in task-irrelevant dimensions [8–10], which is referred to as the minimum intervention principle [11]. The minimum intervention principle is closely related to the concept of optimal feedback control [11], which can explain larger movement variability in task-irrelevant dimensions (e.g., mid-flight in goal-directed arm-reaching movements) and smaller movement variability in task-relevant dimensions (e.g., movement initiation and termination in goal-directed arm-reaching movements). The minimum intervention principle works efficiently to achieve planned movements while minimizing motor cost (e.g., metabolic cost or amplitude of motor commands) and solving the redundancy problem.

Overall, there are feasible solutions to redundancy problems. A solution is to construct spatial and temporal modules. Another solution is to decompose time-varying and multidimensional motions into task-relevant and task-irrelevant components after finding the relationships between motion and task achievements. Evidence from several studies supports these possibilities.

However, the relationships among the spatial module, temporal module, and task-relevant component remain unclear as discussed in a previous study [12]. A common method used to extract spatial and temporal modules is matrix decomposition. Previously, researchers have utilized methods of matrix decomposition, such as principal component analysis (PCA) to analyze joint angle data [5] or nonnegative matrix decomposition (NNMF) [13] to analyze muscle activity data [3, 4, 6]. Because singular value decomposition (SVD) is theoretically the same framework as PCA, SVD can be useful in analyzing joint angle data. Matrix decomposition (e.g., PCA, SVD, or NNMF) allows us to analyze prominent spatial and temporal information (i.e., spatial and temporal modules) to reconstruct original motion data with low-dimensional components; nevertheless, these methods do not consider task-relevant components. A common method used to extract task-relevant components is the uncontrolled manifold (UCM) [8]. For example, UCM enables us to decompose motion data into relevant and irrelevant components of the motion of the center of the body mass in sit-to-stand motion [8]. The decomposition procedure consists of forward kinematics to model the region of interest (ROI), calculation of the averaged joint angles across trials in each time frame, linearizing the forward kinematics around the averages, and calculation of relevant and irrelevant directions to the ROI. There is limited information regarding the spatial and temporal modules in the framework of UCM.

The relationship between spatial modules and task-relevant components has been discussed. We can evaluate the rank of each spatial and temporal module based on the contribution to the reconstruction of the original data. In extreme cases, only higher-rank spatial modules are considered important, and lower-rank modules are neglected. Nevertheless, in reproducing grasping and using various familiar tools by the right hand, both higher-rank and lower-rank modules possess task-related information [14]. In grasping, using tools, and speaking American sign language, both higher-rank and lower-rank modules retain information to separate words [15]. Our earlier study also demonstrated the embedding of task-relevant information in spatial and temporal modules independent of the rank of the modules [10]. Based on these results, we can assume that task-relevant information is embedded into spatial and temporal modules regardless of their ranks; thus, the relationship between the modules and task-relevant components is still unclear.

The relationship among spatial modules, temporal modules, and their task relevance has also been discussed. To confirm whether spatial and temporal modules are commonly utilized in different tasks, it is possible to compare spatial and temporal modules between two or more tasks [16]. A previous study measured all muscles relevant to force-production tasks and discussed the relevance of the modules to task performance [17]. Interestingly, a recent study revealed that extracted spatial and temporal modules are related to not only motor execution but also perceptual inference [18]. From a control-theoretical viewpoint, a framework of hierarchical optimal feedback control provides a hint regarding how spatial modules are related to task achievements [19]. From the perspectives of data-driven methods, tensor decomposition or its variants are effective methods used to explore the relationship among spatial modules, temporal modules, and their task relevance [7, 20, 21]. While analyzing tensor data, including spatial, temporal, and task information, these methods enable us to extract spatial modules and temporal modules and determine how the modules are recruited in each task condition. For example, [7], some spatial and temporal modules were highly recruited as the locomotion speed increased independent of either walking or running. Some spatial and temporal modules were recruited only while walking or running. Although these methods are effective in determining the relationships among spatial modules, temporal modules, and their task relevance, they do not extract task-relevant components. Therefore, tensor decomposition and its variants focus on the reconstruction of original motion data rather than the extraction of task-relevant components while modeling the relationship between motion and performance (e.g., forward kinematics in considering the center of mass in locomotion). Thus, the relationships among spatial modules, temporal modules, and task-relevant components remain unclear.

**Fig 1:**
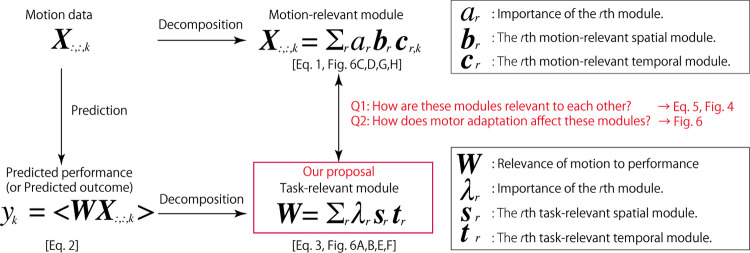
Diagram of our proposal. In conventional studies, motion data were decomposed into motion-relevant spatiotemporal modules (the upper part). In our study, we calculated relevant modules to predict the task outcome (the lower part). We assume that a linear relationship exists between motion and performance (the lower-left part), which has been validated in previous studies [10, 23, 24]. The bracket in the lower-left part indicates the inner product of the matrices ***W*** and ***X***_:,:,*k*_.

Here, we propose a framework to extract task-relevant spatial and temporal modules (see Fig 1 for a summary of our proposal and analyses). In contrast to conventional modules estimated based on the reconstruction power of the original data, our framework enables us to extract modules relevant to task achievements. Hereafter, the current study refers to conventional modules as motion-relevant modules. Similarly, we refer to the modules relevant to task achievements as task-relevant modules. A comparison of motion-relevant and task-relevant modules provides insight into the relationship between motion-relevant modules and task-relevant components. First, our framework is based on ridge regression [22] to predict task performance based on time-varying multidimensional motion data [10, 23, 24]. A ridge regression allows us to quantify how motion data in each time frame in each body part are relevant to predicting task performance. Thus, a ridge regression provides the task relevance of each motion data point while mixing spatial and temporal aspects rather than separating spatial from temporal aspects. Because motion-relevant modules possess spatial and temporal information independently, it is difficult to compare task-relevant components to motion-relevant modules via a ridge regression alone. Thus, the current study extracted task-relevant spatial and temporal modules by utilizing SVD, which permits us to dissociate spatial information from temporal information. We rely on SVD for the following reasons. First, we focus on joint-angle data, including both positive and negative values after standardization. Second, SVD enables us to extract the same spatial and temporal modules as PCA, a major method used to extract motion-relevant modules from joint angle data [5]. Third, SVD enables us to extend theoretical analyses while simultaneously focusing on both spatial and temporal aspects. Therefore, NNMF is not effective in our case because NNMF is a sophisticated tool used to analyze data that include nonnegative values (e.g., muscle-activity or neural-activity data). Thus, by utilizing SVD, the current study analytically evaluates the relationship between task-relevant and motion-relevant modules.

The current study also examines the function of task-relevant modules while focusing on motor adaptation [26–32], where motion is modified in a trial-to-trial manner under the existence of motor error between targeted and actual motor outcomes. In the paradigm of motor adaptation, the relationship between motion and the task outcome changes by perturbation, such as unpredictable force applied to some body parts [25, 26] or unpredictable changes in visual information reflecting the motion outcome [27–29]. The features of motor adaptation are frequently examined in arm-reaching movements while focusing on the hand position or force generated by the hand [25–29]. Although several features of motor adaptation have revealed arm-reaching movements, how to modify motor outcomes in whole-body movements with hundreds of DoFs is unclear. Several studies have reported adaptation-dependent modulations of motion-relevant temporal modules [30–32]. Nevertheless, the task relevance of motion-relevant modules is unclear. We speculate that a function of task-relevant modules is to modify motor outputs while reducing the number of DoFs, i.e., we speculate that task-relevant modules may show adaptation-dependent modulations. To validate this prediction, the current study examines motion-relevant modules in a motor adaptation paradigm of whole-body movements [10].

## Results

### Detection of task-relevant spatial and temporal modules

The current study proposes a method to extract task-relevant spatiotemporal modules via a ridge regression and singular value decomposition (SVD). All detailed calculations are provided in the Methods section. Additionally, Fig 1 shows the purpose and summary of the following calculations.

First, we clarified a conventional method to extract motion-relevant modules from motion data ***X***_:,:,*k*_∈ℝ^[*I*,*J*]^ at the *k*th trial, where *I* and *J* indicate the number of joints and time frames to be considered. Throughout our study, all motion and performance data were standardized such that the mean and standard deviation were 0 and 1, respectively. In the motion data, the standardization procedure was applied to each joint angle across all time frames and trials. The motion data are decomposed as follows:

$$X_{:,:,k}=\overset{R_{m}}{\underset{r=1}{\sum }}a_{r}b_{r}c_{r,k}^{T},$$ (1)

where *R*_m_ is the number of modules, *a*_*r*_≥0 is the relevance of the *r*th motion-relevant modules to reconstruct ***X***_:,:,*k*_ ($a_{1}\geq a_{2}\geq \cdots \geq a_{R_{m}}\geq 0$), ***b***_*r*_∈ℝ^[*I*,1]^ is the *r*th motion-relevant spatial module ($b_{i}^{T}b_{j}=0$ [*i*≠*j*] and $b_{i}^{T}b_{i}=1$), ***c***_*r*,*k*_∈ℝ^[*J*,1]^ is the *r*th motion-relevant temporal coefficients ($c_{i,k}^{T}c_{j,k}=0$ [*i*≠*j*] and $c_{i,k}^{T}c_{i,k}=1$), and $c_{r,k}^{T}$ is the transpose of ***c***_*r*,*k*_. Because ***c***_*r*,*k*_ depends on the trial, we refer to ***c***_*r*,*k*_ as temporal coefficients rather than temporal modules. In these calculations, the motion-relevant spatial and temporal components are assumed to be invariant and variant across trials, respectively (see the Methods section for the opposite case).

Second, we propose a method to extract task-relevant modules. In preparation, we clarified a method to estimate the relevance of motion to task performance. By using multiple joints and time-varying motion data, we estimated the relevance of the motion data ***X***_:,:,*k*_ to performance data *d*_*k*_∈ℝ at the *k*th trial (*k* = 1,…,*K*). To estimate the relevance, our earlier studies demonstrated the effectiveness of a ridge regression rather than some nonlinear regression techniques [10, 23, 24]. A ridge regression allows us to estimate the relevance of motion to performance data ***W***∈ℝ^[*I*,*J*]^ while minimizing prediction error in the presence of observation noise (see Methods for details). Through a ridge regression, the relevance of motion to performance is written as follows:

$$y_{k}=\langle W,X_{:,:,k}\rangle +const.=\overset{I}{\underset{i=1}{\sum }}\overset{J}{\underset{j=1}{\sum }}X_{i,j,k}W_{i,j}+const.,$$ (2)

where *y*_*k*_ indicates the predicted performance, ⟨***W***, ***X***_:,:,*k*_⟩ indicates the inner product of ***W*** and ***X***_:,:,*k*_, and const. denotes a byproduct constant value independent of ***X*** and ***W*** in the standardization processes. Notably, the relevance of motion to performance, ***W***, enables us to calculate the task-relevant and task-irrelevant motion components in a data-driven manner [10]. Thus, we can consider the relevance of motion to performance ***W*** to include equivalent information to task-relevant and task-irrelevant motion components. The predicted performance is determined to minimize the prediction error between the actual and predicted performance. *W*_*i*,*j*_ indicates how the *i*th joint angle at the *j*th time frame is relevant to performance.

After evaluating the relevance of motion to performance ***W***, we extracted task-relevant modules. SVD enabled us to obtain the modules by decomposing ***W*** as follows:

$$W=\overset{R_{p}}{\underset{r=1}{\sum }}\lambda_{r}s_{r}t_{r}^{T},$$ (3)

where *R*_p_ is the number of modules, *λ*_*r*_≥0 is the relevance of the *r*th task-relevant spatiotemporal module to reconstruct ***W*** ($\lambda_{1}\geq \lambda_{2}\geq \cdots \geq \lambda_{R_{p}}\geq 0$), ***s***_*r*_∈ℝ^[*I*,1]^ is the *r*th task-relevant spatial module ($s_{i}^{T}s_{j}=0$ [*i*≠*j*] and $s_{i}^{T}s_{i}=1$), and ***t***_*r*_∈ℝ^[*J*,1]^ is the *r*th task-relevant temporal module ($t_{i}^{T}t_{j}=0$ [*i*≠*j*] and $t_{i}^{T}t_{i}=1$). Because ***W*** is equivalent to task-relevant and task-irrelevant components, as mentioned above, the task-relevant spatial and temporal modules include spatial and temporal information embedded in task-relevant and task-irrelevant components.

While utilizing task-relevant modules, the relevance of motion to performance can be written as follows:

yk=⟨W,X:,:,k⟩+const.=∑r=1RpλrsrX:,:,ktrT+const.=∑r=1Rpy^k,r+const.. (4)

If ***X***_:,:,*k*_ possesses similarities to ***s***_*r*_ in spatial aspects and ***t***_*r*_ in temporal aspects, the bilinear calculation in Eq 4, $s_{r}X_{:,:,k}t_{r}^{T}$ yields a large absolute value of performance. If ***X***_:,:,*k*_ does not possess similarities to ***s***_*r*_ in spatial aspects and ***t***_*r*_ in temporal aspects, the bilinear calculation yields a small absolute value of performance. Both the *r*th task-relevant spatial and temporal modules yield a fragment of predicted performance y^k,r. The magnitude of y^k,r reflects the contribution of the *r*th task-relevant spatiotemporal module to the reconstruction of the original predicted performance.

Although it was possible to find the similarity of equation forms between Eqs 1 and 3, the purposes of the calculations were different. The motion-relevant modules were determined based on the explanatory power of the original motion data ***X*** [3–7] (Eq 1). In contrast, the task-relevant modules were determined based on the explanatory power of the relevance of motion to performance ***W*** (Eq 3) rather than the original motion data ***X***. Despite the difference in their purposes, the similarity of equation forms between Eqs 1 and 3 enable us to directly compare the motion-relevant modules and task-relevant modules.

Finally, we analytically considered the relationship between the task-relevant and motion-relevant modules. The vectorized relevance of motion to performance vec(***W***)∈ℝ^[1,*IJ*]^ can be written as follows (see the Methods section for details):

$$vec(W)=\overset{R_{st}}{\underset{r=1}{\sum }}f(\omega_{r})g_{v_{r}}(Cor(x,d))v_{r},$$ (5)

where Cor(***x***, *d*) denotes the correlation coefficient between motion data ***x*** = (***x***_1_, ***x***_2_, …, ***x***_*K*_) (***x***_*k*_ = vec(***X***_:,:,*k*_)) and performance data *d* = (*d*_1_, *d*_2_, …, *d*_*K*_), $g_{v_{r}}(Cor(x,d))$ indicates a linear function of Cor(***x***, *d*) whose coefficients depend on motion-relevant spatiotemporal module ***v***_*r*_, *ω*_*r*_≥0 indicates how ***v***_*r*_ contributes to reconstructing the original motion data, and *f*(*ω*_*r*_) indicates a monotonically decreasing function of *ω*_*r*_. The motion-relevant spatiotemporal module ***v***_*r*_ can be calculated by applying SVD to the vectorized motion dataset as follows: $x_{k}=\overset{R_{st}}{\underset{r=1}{\sum }}\omega_{r}u_{k,r}v_{r}$, where *R*_st_ is the number of modules, *ω*_*r*_≥0 is the relevance of the *r*th module used to reconstruct ***x***_*k*_, *u*_*k*,*r*_ denotes how ***v***_*r*_ is related to the *k*th trial, and ***v***_*r*_ is the *r*th motion-relevant spatiotemporal module. Notably, ***v***_*r*_ includes both spatial and temporal information in a mixed manner in contrast to an independent manner such as the motion-relevant (Eq 1) and task-relevant modules (Eq 3).

Through Eq 5, the relevance of motion to performance was determined based on the motion-relevant spatiotemporal modules $v_{1},\cdots ,v_{R_{st}}$) while weighting each module using Cor(***x***, *d*). Cor(***x***, *d*) indicates the correlation between motion and performance, which allows us to interpret vec(***W***) as the relevance of motion to performance. After the rearrangement of vec(***W***) as matrix ***W***, SVD provided the task-relevant spatial and temporal modules (Eq 3). Thus, the task-relevant modules consist of the motion-relevant modules weighted by the correlation between each module and performance, *g*_*r*_(Cor(***x***, *d*)), and the relevance of each module to reconstructing the original motion data, *f*(*ω*_*r*_). Additionally, Eq 5 indicates a nonlinear relationship between the task-relevant modules ***W*** and motion-relevant modules ***v*** because the multiplication of $g_{v_{r}}(Cor(x,d))$ by ***v***_*r*_ has squared forms of ***v***_*r*_. Although the motion-relevant modules were extracted while focusing only on the reconstruction of the original motion data, the task-relevant modules were determined while considering how motion is related to task performance.

After revealing the relationship between the motion- and task-relevant modules via Eq 5, we examined the function of the task-relevant modules. A perspective regarding Eq 4 was that the task-relevant modules determine the predicted outcomes (i.e., *y*_*k*_) based on planned motion (i.e., ***X***_:,:,*k*_). This function likely enables us to plan appropriate motion to achieve the targeted motion. To validate this speculation, we examined how motor adaptation affected the task-relevant modules. In the paradigm of motor adaptation, we apply unpredictable perturbation to motor output and cause error between the targeted and actual movements [25–32]. To overcome the error, the motor output should be modulated appropriately. If task-relevant modules are related to planning motion to achieve the targeted motion, the modules should show adaptation-dependent modulations.

To explore adaptation-dependent modulations of task-relevant modules, the current study applied our framework to motor adaptation with multiple joints and time-varying motion. Specifically, we examined the motor adaptation of jump motion [10]. Notably, our framework can be applied to a variety of situations, such as throwing a ball or dart to estimate the endpoint [23, 24], jumping to estimate the jumping height or length [10], locomotion to estimate the speed and mode (e.g., [7]), or arm-reaching movements to estimate the generated force at the hand (e.g., [25, 26]).

### Experimental data

**Fig 2:**
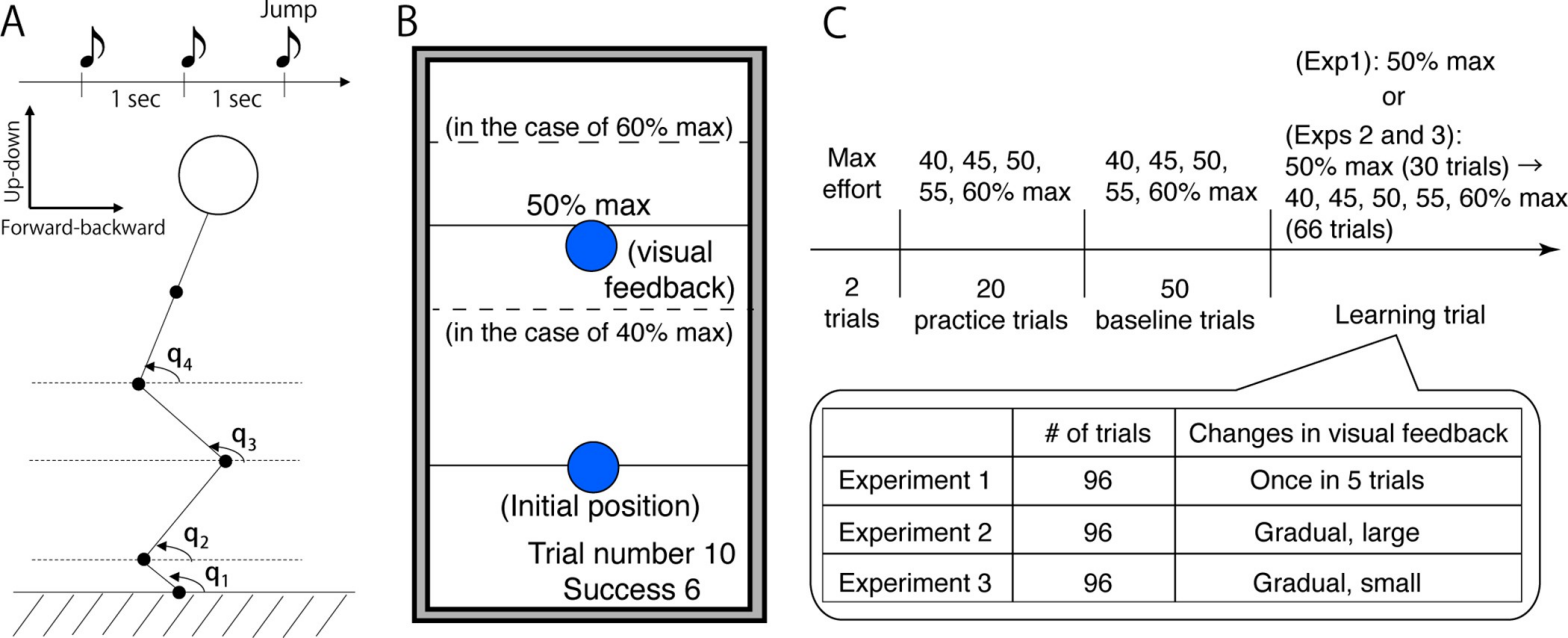
Experimental setting (revised from [10]). (A): One sec after the target height was determined, three beeps were presented. The subjects performed a vertical jump at the timing of the third beep. The interbeep interval was 1 sec. We measured four joint angles, toe (*q*_1_), ankle (*q*_2_), knee (*q*_3_), and hip (*q*_4_). The jumping height was determined based on the position of a marker attached to the back. (B): Instruction and visual feedback on the monitor. The blue cursor indicates the back position. At the beginning of each trial, the blue cursor was displayed on the horizontal solid black line in a lower part of the monitor. Simultaneously, the horizontal solid black line was visible to indicate the target height in an upper part of the monitor. The position of the target line changed depending on the target height (panel B provides examples of when the target height was 40% [the horizontal dotted black line was located lower than the 50% target line] and 60% [the horizontal dotted black line was located higher than the 50% target line]). One sec after the initial presentation, the blue cursor disappeared until the subjects completed the vertical jump. After each vertical jump, the blue cursor was visualized at the jump height without false visual feedback. With false visual feedback, the cursor position was displayed higher or lower than the jumping height depending on the types of false visual feedback. (C): Settings of the target height and false visual feedback. We analyzed both baseline and learning trials.

The subjects performed goal-directed vertical jumps while crossing their arms in front of their trunks (N = 13, Fig 2A). Before the main experiments, the subjects executed a vertical jump with maximum effort while being allowed to use arm swings (Fig 2C). The target height throughout the main experiments was based on the maximum height of each subject, i.e., 40% max, 45% max, 50% max, 55% max, or 60% max. At the beginning of each trial, the subjects were instructed to stand while crossing their arms in front of their trunks, and the target height was displayed (Fig 2B). One second after the target display, three beeps sounded with a one-second interval. The subjects were instructed to jump at the timing of the third beep to control the jumping height to match the target height. The jumping height was measured based on a motion capture marker attached to the back. Subtracting the height at the standing position from the maximum jumping height allowed us to determine the jumping height in each trial. After one vertical jump, the subjects were visually informed of their jumping height, which informed them whether they performed the jumping motion well.

**Fig 3:**
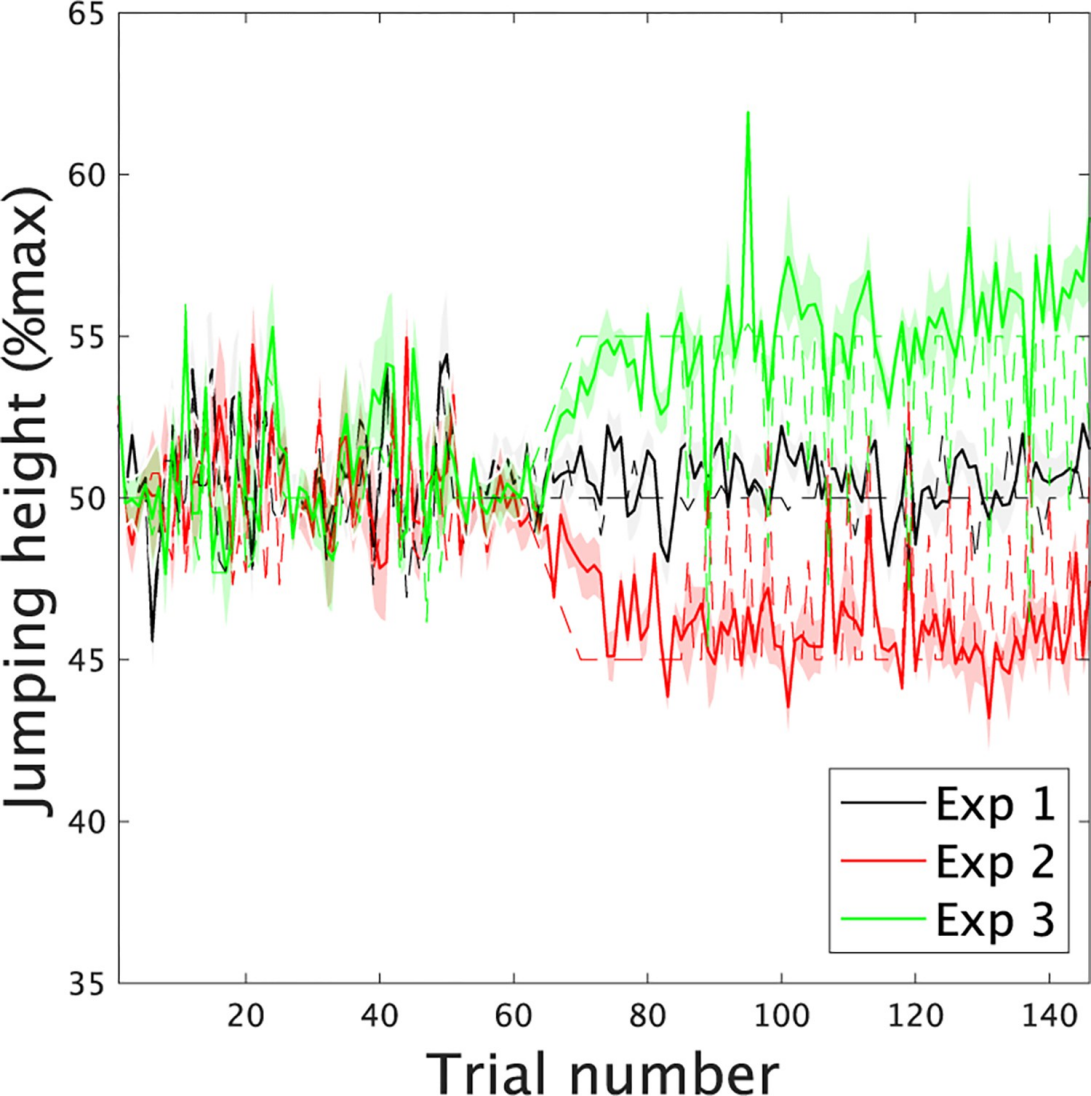
Learning curves in Experiments 1, 2, and 3. Solid black, red, and green lines indicate the mean actual jumping height (%Max) across the subjects in Experiments 1, 2, and 3, respectively. The dotted black, red, and green lines denote the mean desired jumping height across the subjects while considering the target height and false visual feedback (%Max). The black, red, and green shaded areas demonstrate the standard error of the mean of the actual jumping height.

In the learning trials (Fig 2C), we changed the visual feedback of the jumping height to induce motor adaptation. In one day, the changes in visual feedback were inserted once in five trials to confirm the influence of visual feedback on the jumping height (Experiment 1 in Fig 2C). On another day, the informed jumping height was gradually set to be higher than the actual jumping height, which induced motor adaptation to perform a small jump (Experiment 2 in Fig 2C). On another day, the informed jumping height was gradually set to be smaller than the actual jumping height, which induced motor adaptation to perform a large jump (Experiment 3 in Fig 2C). The same subjects participated in the three experiments but not on consecutive days. The order of Experiments 2 and 3 was counterbalanced across the subjects. As a result of Experiment 1, we confirmed motor adaptation via falsified visual feedback [10]. Fig 3 summarizes the jumping height under falsified visual feedback, confirming that motor adaptation occurred in our experiments. All experimental settings and some data were reported in our previous study [10].

The current study focused on the relationship between the jumping height and temporal variations in four joint angles, namely, toe, ankle, knee, and hip angles (Fig 2A), for 500 msec before release (i.e., 60 time frames with a 120-Hz measurement). Hereafter, we refer to jumping height as performance. A ridge regression allows us to estimate the relationship between the motion data and performance with prediction error 0.196±0.0168 (mean ± standard error of mean [s.e.m.] across all subjects and experiments) (Eq 2). If there was no relationship between the motion data and performance, the prediction error would be 1. If there was a perfectly clear relationship between the motion data and performance, the prediction error would be 0. In our setting, a ridge regression allows us to predict performance with an accuracy of approximately 80% based on data of 60 time frames and four joint angles.

### Task-relevant and motion-relevant modules in motor adaptation experiments

**Fig 4:**
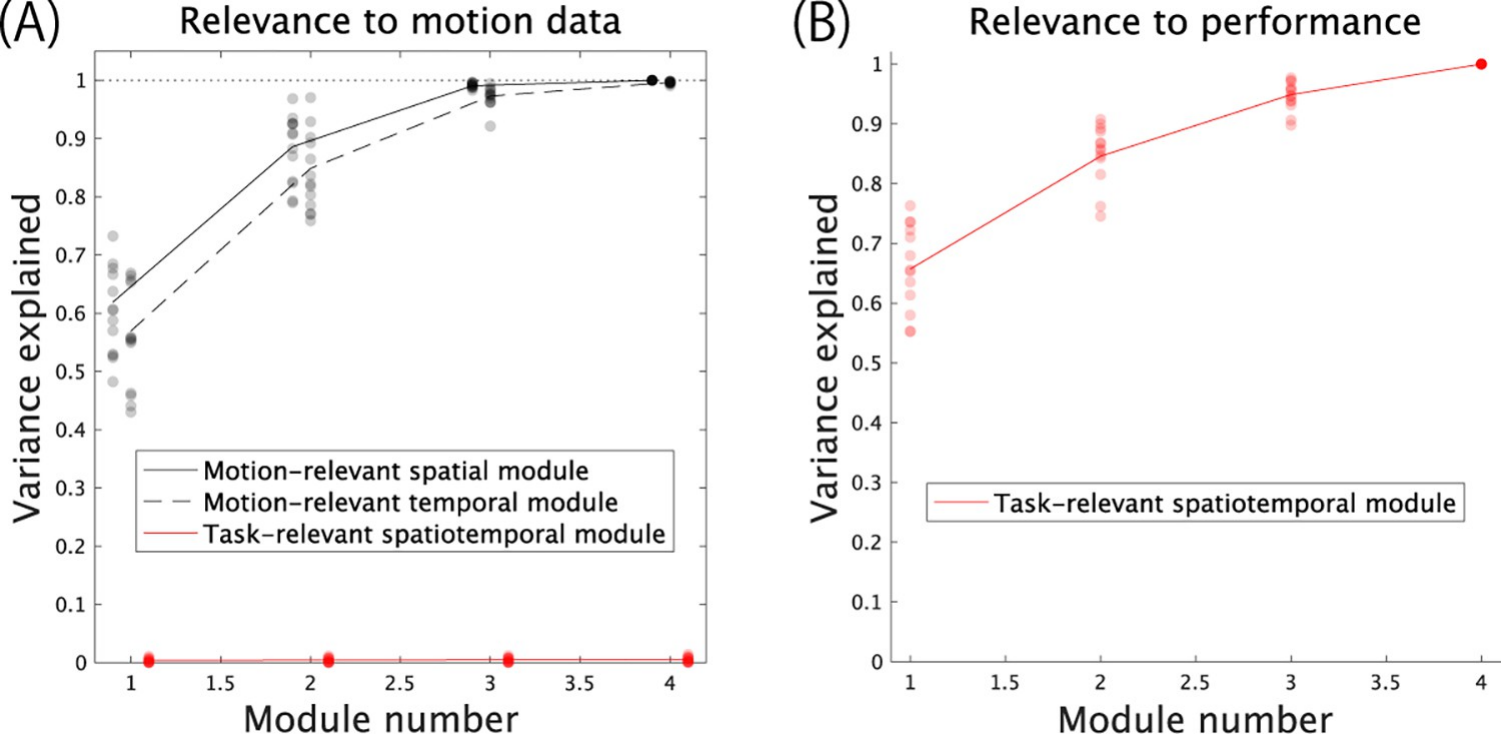
Relevance of task- and motion-relevant modules to motion data and performance. (A): The proportion of variance in the original motion data explained by motion-relevant spatial modules (solid black line), motion-relevant temporal modules (dotted black line), and task-relevant spatiotemporal modules (solid red line). Detailed explanations of the proportion of variance in the original motion data explained by these modules are provided in the Methods section. The horizontal axis denotes the number of modules used to reconstruct the motion data. Each dot indicates the explained variance of each subject. (B): The proportion of variance in the predicted performance explained by task-relevant spatiotemporal modules. We did not consider motion-relevant modules in this panel because the relevance of these modules to performance was unclear.

Based on time-varying and multiple-joints motion data, we compared task-relevant modules to motion-relevant modules. Although motion-relevant modules prioritize the reconstruction of original motion data, task-relevant modules prioritize the reconstruction of the relevance of motion to performance ***W***, including equivalent information to task-relevant and task-irrelevant motion components. In the framework of motion-relevant modules (Eq 1), the 1st pair of modules explains a larger portion of variance in the original motion data (solid and dotted black lines in Fig 4A). The 2nd-, 3rd- and lower-order modules explain a smaller portion of the variance than the 1st module. In contrast, even the 1st task-relevant module explained few portions of the variance in the original motion data (red solid line in Fig 4A). Combining the 1st to 4th modules still explained minimal variance. In contrast, the 1st task-relevant spatiotemporal module explained a larger portion of the predictive power to predict performance from time-varying motion data (Fig 4B). The other lower-order modules played roles in adding predictive power to the 1st module. Because the task-relevant modules were determined by a nonlinear function of motion-relevant modules (Eq 5), there were large gaps between the two types of modules.

**Fig 5:**
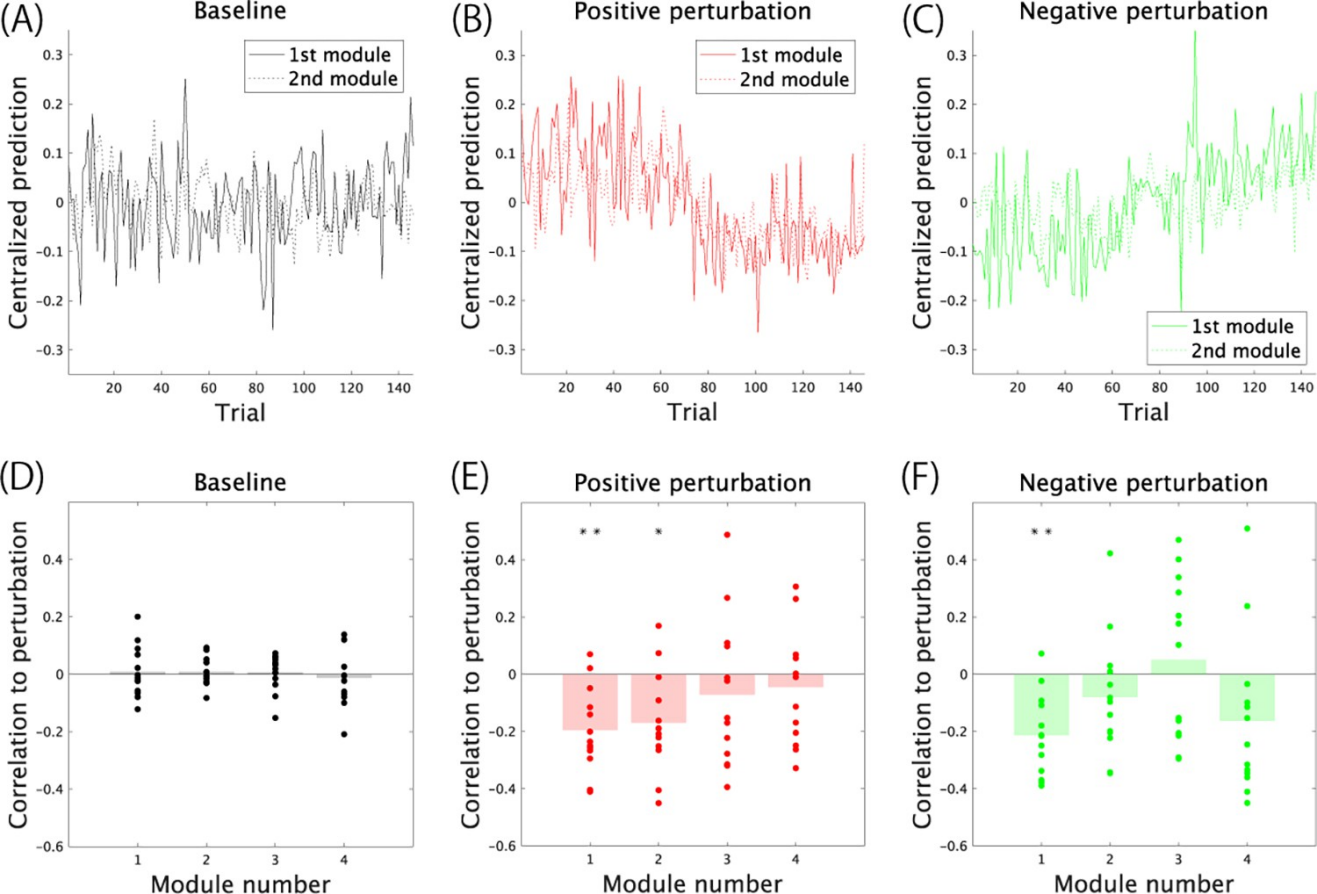
The relevance of each task-relevant module to performance prediction. (A-C): Predicted performance by the 1st and 2nd task-relevant spatiotemporal modules in Experiment 1 (panel A), Experiment 2 (panel B), and Experiment 3 (panel C). All performance values were centralized by subtracting *C* in Eq 4. Solid and dotted lines denote the predicted performance by the 1st and 2nd modules, respectively. (D-E): The correlation coefficients between the applied perturbation and predicted performance by each task-relevant module. Because the subjects needed to compensate for the perturbation to move the cursor position onto the target line, the correlation was negative if the predicted value behaved as if it compensated for the perturbation. Double and single asterisk(s) indicate p < 0.01 and p < 0.05, respectively, according to a t test regarding whether the calculated correlation in all subjects was consistently and significantly different from 0.

Then, we investigated the predicted performance values given by each task-relevant spatiotemporal module, y^k,r, in Eq 4 (Fig 5). The black color in the bar graphs and solid line plots denotes the values in Experiment 1 with no consistent motor adaptation (Fig 5A and 5D). The red and green colors in the bar graphs and solid line plots indicate the values in Experiments 2 and 3 with consistent modulations of the jumping height through environmental changes (Fig 5B, 5C, 5E, and 5F). In Experiment 2, denoted by the red color (Fig 5B and 5E), the subjects performed vertical jumps with a lower jumping height than the target height due to motor adaptation (Fig 3). In Experiment 3, denoted by the green color (Fig 5C and 5F), the subjects performed vertical jumps with a higher jumping height than the target height (Fig 3). The solid and dotted lines in Fig 5A–5C indicate the predicted performance based on the 1st and 2nd task-relevant spatiotemporal modules, respectively. In the black solid and dotted lines or in Experiment 1 (Fig 5A), there was no tendency in the predicted performance because the applied environmental changes or perturbations did not have any tendency. We also examined the correlation between the applied perturbation and the predicted performance by each task-relevant module y^k,r. In motor adaptation, the subjects needed to compensate for the perturbation. When the applied perturbation was negative (i.e., informed that the jumping height was smaller than the actual jumping height), the subjects should jump higher than at baseline (i.e., predicted performance should be positive in Fig 5A–5C). Thus, the correlation between the applied perturbation and y^k,r should be negative if y^k,r represents performance modulated by motor adaptation. We examined whether the correlation was significant based on a t test of the correlation value in all subjects. If there was a consistent correlation across the subjects, the p value in the t test should be smaller than some criteria (e.g., p < 0.05 or 0.01). Along with these findings, there was no significant correlation between the applied perturbation sequence and predicted performance via the modules in Experiment 1 (p > 0.5018 [uncorrected], Fig 5D).

In the red solid and dotted lines or in Experiment 2 (Fig 5B), there were tendencies in both the 1st and 2nd task-relevant spatiotemporal modules, i.e., the predicted values were larger in the former and smaller in the latter trials. There were also significant correlations between the predicted values and perturbation in the 1st and 2nd task-relevant modules (p = 0.0025 [corrected] in the 1st module and p = 0.0215 [corrected] in the 2nd module, Fig 5E). In contrast, in the green solid and dotted lines or in Experiment 3 (Fig 5C), there were tendencies only in the 1st task-relevant spatiotemporal module, i.e., the predicted values were smaller in the former and larger in the latter trials. There was also a significant correlation between the predicted values and perturbation in the 1st task-relevant module (p = 0.0010 [corrected], Fig 5F). The 1st task-relevant module reflected motor adaptation in Experiments 2 and 3. In contrast, the relevance of the 2nd task-relevant module depended on the task settings.

**Fig 6:**
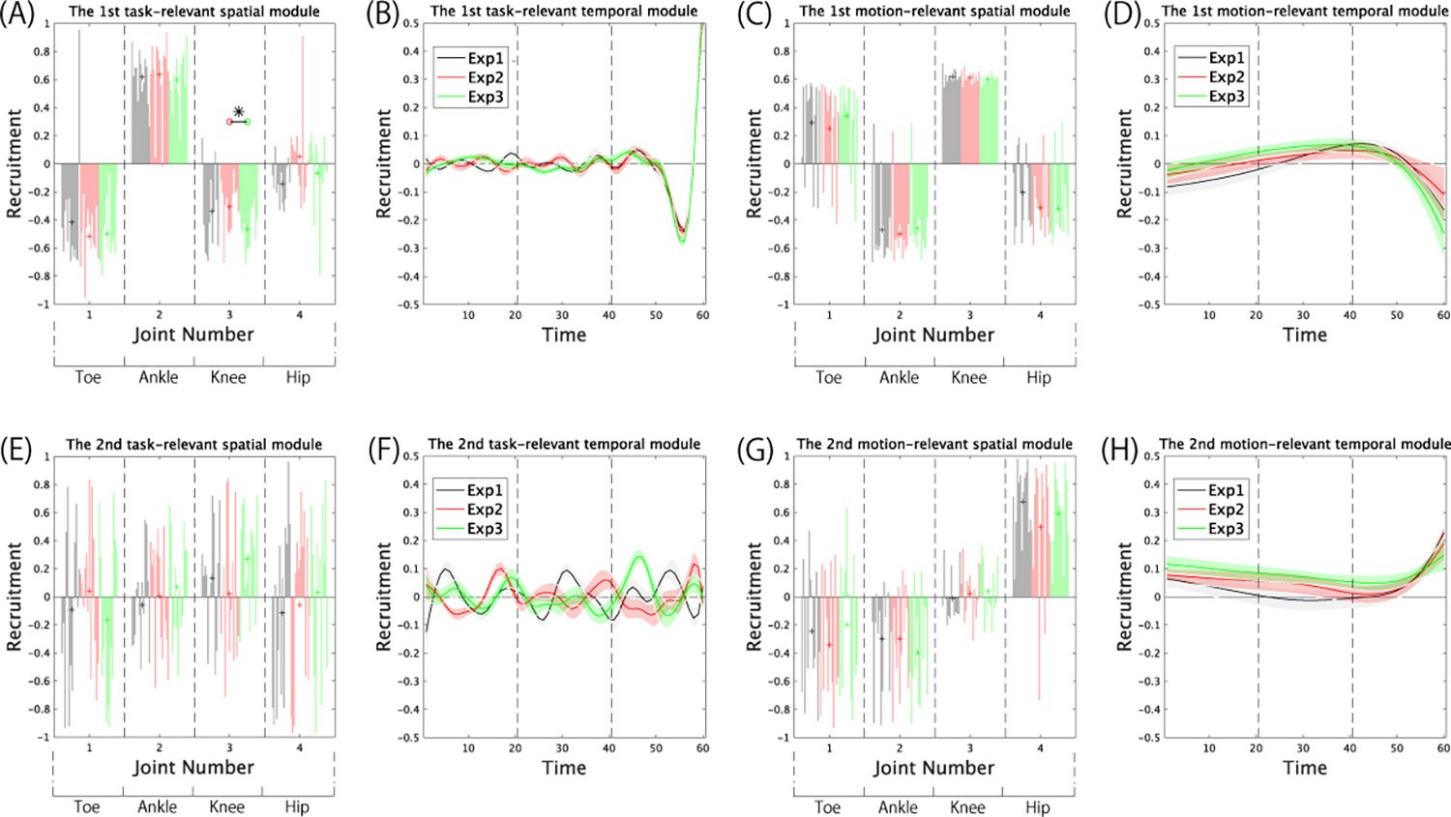
Task- and motion-relevant modules in Experiments 1, 2, and 3. All modules were normalized such that each norm equaled 1. (A): The 1st task-relevant spatial module. Joint numbers 1, 2, 3, and 4 indicate the toe, ankle, knee, and hip angles, respectively. Black, red, and green colors indicate the modules in Experiments 1, 2, and 3, respectively. Each bar graph refers to the module of each subject. For example, the left-most black bar in joint number 1 indicates the recruitment of the toe angle in the 1st task-relevant spatial module. The left-most black bar in joint number 1 is associated with the black left-most bar in joint numbers 2, 3, and 4 to construct the 1st task-relevant spatial module in subject #1. Each cross demonstrates the average across subjects. The asterisk indicates a significant difference (p = 0.0113) based on Tukey’s comparison test. (B): The 1st task-relevant temporal module. Black, red, and green solid lines indicate the modules in Experiments 1, 2, and 3 averaged across the subjects, respectively. Each shaded area denotes the standard error of the mean. To perform the one-way ANOVA and Tukey’s comparison test, we divided the temporal module into three phases denoted by the vertical dotted black lines. After the division, the value in the module was averaged within each phase and subject, and statistical tests were performed. (C, D): The 1st motion-relevant spatial and temporal module. (E, F): The 2nd task-relevant spatial and temporal module. (G, H): The 2nd motion-relevant spatial and temporal module.

Finally, we investigated adaptation-dependent modulations of motion-relevant and task-relevant modules. In the paradigm of motor adaptation, we changed the relationship between the actual and informed jumping height while adding perturbation to the feedback information (see Methods for details). In this setup, the subjects need to change how they predict motion outcomes compared to the situations without any perturbation. If task-relevant modules play a role in predicting motion outcomes, they should show more significant adaptation-dependent modulations than motion-relevant modules. Fig 6 demonstrates the 1st and 2nd task-relevant and motion-relevant spatial and temporal modules and their adaptation-dependent modulations that were examined via a comparison of Experiments 2 and 3. Among the features demonstrated in Fig 6, there was one significant main group effect (i.e., the modulation depending on either Experiment 1, 2, or 3) in the 3rd component of the 1st task-relevant spatial module (i.e., knee joint angle, Fig 6A, F = 3.6642, p = 0.0409). There was no main group effect in the other features (p > 0.273 in the other features in the task-relevant spatiotemporal modules and p > 0.0917 in the motion-relevant spatiotemporal modules). Based on a multiple comparison with Tukey’s comparison test, there was a significant difference between Experiments 2 and 3 in the 3rd component of the 1st task-relevant spatial module (p = 0.0113). Regarding the other features, there was no difference in the results of Tukey’s comparison test (p > 0.240 in the other features in the task-relevant spatiotemporal modules and p > 0.0953 in the motion-relevant spatiotemporal modules). These results indicate that adaptation-dependent modulations were observable in the 1st task-relevant spatial module rather than the task-relevant temporal modules and the motion-relevant spatiotemporal modules.

## Discussion

The current study proposed a framework with which to extract task-relevant modules via a ridge regression and SVD (Eqs 2–4). The task-relevant modules originated from a nonlinear function of motion-relevant spatiotemporal modules based on their relevance to task performance (Eq 5). Due to the nonlinear relationship between the motion-relevant and task-relevant modules, there were few similarities (Figs 4 and 6). The motion-relevant modules are prioritized to explain the original motion data (Eq 1, solid and dotted black lines in Fig 4A). In contrast, the task-relevant spatial and temporal modules had limited explanatory power for the original motion data (solid red line in Fig 4A), but these modules explained the relevance of motion to performance (solid red line in Fig 4B). The differences were also evident in the components of each module (Fig 6). In our concept, the task-relevant modules determined the relationship between planned motion and the predicted outcome. Thus, the task-relevant modules were expected to be related to planning appropriate motions to achieve targeted motions with large numbers of DoFs. Under the existence of error between targeted and actual motion, task-relevant modules are modulated to plan motions to achieve targeted motions. Along this concept, there were significant adaptation-dependent modulations in the task-relevant spatial modules rather than the motion-relevant spatiotemporal modules. Summarizing these results, although the task-relevant and motion-relevant spatiotemporal modules are related to each other (Eq 5), they have different features related to motion, predicted performance, and motor adaptation (Figs 4 and 6).

Although some studies have identified possible differences between task-relevant dimensions and motion-relevant spatiotemporal modules [30–32], the relationship between the two concepts remains unclear. In particular, why low-rank motion-relevant modules (that barely reconstruct the original motion data) are relevant to task performance is unclear. The current study proposed a possible answer based on Eq 5, which indicates the relationship between the relevance of motion to performance, ***W***, and motion-relevant spatiotemporal modules, ***v***. If the rank of each motion-relevant module was the only factor determining its relevance to task performance, the contribution of each module to reconstructing the original motion data, *ω*_*r*_, would solely determine the relevance of motion-relevant modules to task performance. However, Eq 5 indicates that the contribution of each module to task performance can be determined by not only the contribution to reconstructing the original data, *ω*_*r*_ but also the weighted sum of the correlation between motion-relevant modules and performance, $g_{v_{r}}(Cor(x,d))$ (the details of $g_{v_{r}}(Cor(x,d))$ are provided in the *Methods* section). Thus, the rank of each motion-relevant module in reconstructing the original motion data does not reflect how each motion-relevant module contributes to task performance; there were large gaps between the motion-relevant and task-relevant modules (Figs 4 and 6). In total, motion-relevant modules are closed concepts within reconstructing the original data that possess features inherent in the human body, such as range of motion in joint angles.

In contrast, task-relevant dimensions somewhat consider the innate features of the human body but primarily focus on the relevance of motion to performance ***W***, which enables us to calculate task-relevant and task-irrelevant motion components [10]. When focusing on the relevance of motion to performance, we should focus on task-relevant dimensions or task-relevant modules rather than motion-relevant modules. Thus, we should use appropriate concepts, e.g., either motion-relevant or task-relevant modules, along with the research purpose.

Consistent with this scenario, our results provide a feasible way to plan appropriate motions with large numbers of DoFs as follows: 1) the CNS extracts motion-relevant spatiotemporal modules [i.e., ***v***_*r*_ in Eq 5 rather than ***b***_*r*_ and ***c***_*r*_ in Eq 1], 2) the CNS determines the correlation between each motion-relevant spatiotemporal module and task outcomes (i.e., these manipulations enable the CNS to estimate the relevance of motion to performance, ***W*,** via Eq 5, 3) The CNS extracts task-relevant modules to reduce the numbers of DoFs [Eq 3], 4) the CNS predicts task outcomes based on planned motion and task-relevant modules [Eq 4], and 5) the CNS determines appropriate motor patterns by imaging various motor repertoires. In this schema, the modulations of task-relevant modules can be related to changes in the correlation between motion-relevant modules and task outcomes in procedure 2. Although the motion-relevant modules differed from the task-relevant modules (Fig 6), this schema reconciles both types of modules and provides a possible implementation for the planning of the appropriate motion in goal-directed situations.

Our adaptation paradigm utilized gain adaptation [27] to induce changes in the motor magnitude or jumping height. In our vertical jump experiments (Fig 2), the task constraint is to perform a vertical jump to match the target height. In the one-dimensional task, gain adaptation could be the only applicable adaptation type. Other types of adaptation include visuomotor rotation [27–29, 33, 34], adaptation in movement direction, force field adaptation [25, 26], or adaptation in the external force. In particular, there are several differences between gain adaptation and adaptation to visuomotor rotation [35]. Different types of adaptation could result in different adaptation-dependent modulations.

We found adaptation-dependent modulation in a component in the 1st task-relevant spatial module (Fig 6A). Modulation was evident between Experiments 2 and 3. In these experiments, the subjects performed lower and higher jumps via motor adaptation. There is a possibility that the difference in jumping height affected the 1st task-relevant spatial module. If this possibility is correct, we should expect invariant task-relevant modules and variant motion data across Experiments 2 and 3. Because the relevance of motion to performance was modeled as *y*_*k*_ = ∑_*i*,*j*_*X*_*i*,*j*,*k*_*W*_*i*,*j*_, the difference in motion data with the same task relevance resulted in different jumping heights. In contrast to this speculation, the task-relevant modules differed between Experiments 2 and 3 (Fig 6A). In both Experiments 2 and 3, the subjects performed diverse jumping heights from 40% to 60% Max target height. If the jumping height affected the relevance of motion to performance *W*, the relevance should change within each experiment appropriate for lower and higher target heights. In contrast to this speculation, *W* was constant, and the difference in motion data *X* in each trial modulated jumping height within each experiment (Fig 5). Thus, adaptation rather than the difference in jumping height possibly affected the relevance of motion to performance ***W***, i.e., task-relevant spatial and temporal modules.

A possible explanation for why a linear regression works well is an analogy with the motor primitive framework. The motor primitive framework can successfully model motor adaptation [28, 36, 37]. In this framework, a nonlinear motor command *u* is determined as the linear weighted sum of nonlinear neural activities *A*: *u* = ∑_*i*_*τ*_*i*_*A*_*i*_. The weighted coefficients *τ*_*i*_ are modified to minimize the movement error between the actual and desired movement performance. When *A*_*i*_ is a nonlinear function of the desired movement and appropriately high-dimensional, appropriate linear combinations of nonlinear neural activities can generate desired motor commands, which has been theoretically validated in the framework of a basis function network [38]. In our case, the motion data ***X*** can be a nonlinear function of movement performance because body dynamics are nonlinear, and movements are performed to achieve task requirements. Additionally, the motion data are appropriately high-dimensional when we use multiple time frames rather than only time frames at release timing. Thus, an appropriate linear summation ***Xw*** while using multiple time frames could predict the actual movement performance.

The kinematic data at the release timing should be enough to determine performance in ballistic tasks. In our case, we expected to model jumping height *h* by parabola using release position *p* and release velocity *v* as $h=p+\frac{v^{2}}{2g}$, where *g* = 9.8(*m*/*s*^2^) was gravitation acceleration; nevertheless, the prediction error was 0.382±0.103 in comparison to 0.196±0.0168 by the ridge regression. We calculated *p* and *v* by using forward kinematics. This worse prediction by parabola may be caused by measurement error. Even in the existence of measurement error, a ridge regression works well because a ridge regression is equivalent to a regression while assuming the existence of nonbiased measurement noise [10]. Another possible reason why the ridge regression showed better prediction accuracy than the parabola regression is the number of variables utilized in each framework, i.e., the parabola utilized two variables (i.e., *p* and *v*) and the ridge regression utilized four variables (i.e., toe, ankle, knee, and hip angles) multiplied by the number of time frames. In contrast to this possibility, the temporal variations of each joint angle show correlations, resulting in collinearity, which causes the regression coefficients to be nonvalid [39]. Therefore, a larger number of variables is not always better for predicting performance. A ridge regression is an effective way to overcome collinearity and enabled us to obtain valid regression coefficients [22]. In summary, a ridge regression is an effective way to predict performance under the existence of observation noise and collinearity.

Our approach was a data-driven approach in contrast to modeling the relevance of motion to performance by forward kinematics or parabolas [8, 9]. The strength of the data-driven approach is its applicability even when we do not know the relevance of motion to performance. In our vertical jump setting, it is possible to model the jumping height by a parabola function with the position and velocity of the back position at the release timing. By linear expansion of the parabola function, it is possible to explore task-relevant and task-irrelevant components. This framework is referred to as GEM [9]. In this case, we cannot consider any temporal information. Because the relationship between motion and performance is evident to some degree at the release timing, how motion 10 msec before the release is related to performance is unknown. A data-driven approach enables us to consider these unknown relationships while estimating the relationship based on data. Additionally, the prediction accuracy was better when considering the temporal variation compared to that using parabolic modeling [10]. By considering temporal information, we can obtain both spatial and temporal modules (Eq 4). Furthermore, the data-driven approach allows us to clarify the relationship between motion-relevant and task-relevant modules (Eq 5). However, the data-driven approach requires certain amounts of data to achieve a higher prediction accuracy (cf. [40–42]). With sufficient data, a data-driven approach enables us to consider the relevance of time-varying and multidimensional motion data to performance, task-relevant and task-irrelevant dimensions [10, 24], and task-relevant spatiotemporal modules (Eq 3, Fig 6).

In summary, the current study proposed a framework of task-relevant modules (Eq 3) to reconcile the following two concepts to solve the redundancy problem: motion-relevant modules [1–7] (Eq 1) and task-relevant motion components [1, 8–10]. We analytically derived the nontrivial relationship between task-relevant modules and motion-relevant spatiotemporal modules (Eq 5). Due to the nonlinear relationship, there were large gaps between the task- and motion-relevant modules (Figs 4 and 6). To examine the functional role of task-relevant modules, we examined adaptation-dependent modulations of task-relevant modules. Consistent with our speculation, motor adaptation significantly affected the task-relevant modules rather than motion-relevant modules (Fig 6).

## Methods

### Materials and methods

#### Experimental setup

The detailed experimental setup is provided in our previous publication [10]. The following are the details of the participants, data acquisition, and data preprocessing.

#### Participants

Thirteen healthy volunteers (aged 18–22 years, two females) participated in all our experiments, which were approved by the ethics committee of the Tokyo University of Agriculture and Technology and were performed in accordance with all guidelines and regulations. All participants were informed of the experimental procedures in accordance with the Declaration of Helsinki, and all participants provided written informed consent before the start of the experiments. On the first day, the participants underwent ten practice trials and 160 baseline trials with pseudorandomly changing targets (40%, 45%, 50%, 55%, or 60% of the maximum jumping height) to become familiarized with the experimental setting. On the second, third, and fourth days (not consecutive), they participated in Experiments 1, 2, and 3. The orders of Experiments 2 and 3 were counterbalanced across the subjects. All experimental settings and some data were reported in our previous study [10].

#### Data acquisition and processing

The jumping motions were recorded in 120 Hz using six cameras (Optitrack Flex 13, NaturalPoint Inc., Corvallis, Oregon). Markers were attached to each participant’s back (TV10), right hip joint (femur greater trochanter), right knee (femur lateral epicondyle and femur medial epicondyle), right heel (fibula apex of the lateral malleolus and tibia apex of the medial malleolus), and right toe (head of the 2nd metatarsus). The marker position data were filtered with a 12th-order, 10-Hz zero-phase Butterworth filter using MATLAB 2016a. The joint angles between the right toe and heel (*q*1), right heel and shank (*q*2), right shank and thigh (*q*3), and right thigh and trunk (*q*4) were calculated in the sagittal plane (Fig 1A). Because the current study focused on a vertical jump with the arms crossed in front of the trunk, it was possible to focus only on lower limb and trunk motions. Throughout the current study, we focused on a four-link model of the lower limbs in the sagittal plane. The time of release was detected based on the moment at which the vertical toe position exceeded 10% of the maximum height in each trial. All experimental settings and some data were reported in our previous study [10].

#### Details of the practice trials

During the practice trials (Fig 1C), to inform the subjects of their back position, the cursor position was displayed during preparatory motions, during jumping motions, and after jumping motions. This setting allowed the subjects to become familiar with our experimental setting.

#### Details of the baseline and learning trials

In the baseline and learning trials, the cursor position was visualized before and after the jumping motions. In the baseline trials, the target line was set to either 40%, 45%, 50%, 55%, or 60% of the height at the vertical jump with maximum effort in each subject (Fig 1C). Each target height appeared once within the five trials in a pseudorandom manner.

In the learning trials, in Experiment 1, the target height was fixed to be 50% of the maximum height across the trials. The purpose of Experiment 1 was to assess whether false feedback induced motor adaptation. As described in our earlier study [10], we confirmed that false feedback induced motor adaptation.

In the learning trials in Experiments 2 and 3, the target height was fixed to be 50% in the first 30 trials. We applied false feedback, the magnitude of which gradually increased because these gradually applied perturbations can yield motor adaptation without awareness [29, 33, 34, 43]. The motor adaptation that occurs without awareness enables us to examine motor adaptation with few cognitive factors. In the first 10 of 30 trials, the subjects performed a vertical jump under normal visual feedback. In the following 10 trials, the position of the feedback cursor changed 0.005% per trial, resulting in a 0.05% change in the 20th trial in the learning trials. In Experiment 2, the cursor position was visualized as 0.05% larger than the actual jumping height. The subjects needed to jump 0.05% lower than the height in the baseline trials to position the cursor at the target height. In Experiment 3, the cursor position was visible at a location 0.05% lower than the actual jumping height. In the remaining 10 trials, the magnitude of the false visual feedback was fixed at 0.05% to enable the learning effect to be stable. After 30 trials, the target height was set to either 40%, 45%, 50%, 55%, or 60% of the maximum height once in three trials as a probe trial. These probe trials allowed us to examine whether the subjects adapted to the false visual feedback regarding the target height that differed from the learning target height (i.e., 50% of the maximum height). The target height in the two consecutive trials was set to 50% to allow the subjects to maintain motor adaptation at the trained target height [10].

#### Task-relevant spatiotemporal modules

The current study proposed a way to extract task-relevant spatiotemporal modules. We utilized a ridge regression and singular value decomposition. The following provides the details of the procedures.

Ridge regression: Standardization procedures are indispensable for estimating the relevance of motion to performance $\overset{˜}{W}$ such that $y_{k}=\sum_{i}\sum_{j}x_{i,j,k}{\overset{˜}{W}}_{i,j}$, where *y*_*k*_ is the predicted performance at the *k*th trial, and *x*_*i*,*j*,*k*_ is the standardized motion data for a ridge regression, including the *i*th spatial feature and *j*th temporal feature. Performance data *d*_*k*_ and motion data *x*_*i*,*j*,*k*_ were standardized such that $\frac{1}{K}\sum_{k=1}^{K}d_{k}=0$, $\frac{1}{K}\sum_{k=1}^{K}d_{k}^{2}=1$, $\frac{1}{K}\sum_{k=1}^{K}x_{i,j,k}=0$, and $\frac{1}{K}\sum_{k=1}^{K}x_{i,j,k}^{2}=1$ for all *i* and *j*. Therefore, the averaged value across the trials should be 0, and the trial-to-trial variability should be 1 for all data. For standardization, the original motion data ${\overset{˜}{X}}_{i,j,k}$ were transformed as follows:

$$x_{i,j,k}=\frac{{\overset{˜}{X}}_{i,j,k}-m_{i,j}}{s_{i,j}},$$ (6)

where $m_{i,j}=\frac{1}{K}\sum_{k}{\overset{˜}{X}}_{i,j,k}$ is the mean, and $s_{i,j}=\sqrt{\frac{1}{K}\sum_{k}({\overset{˜}{X}}_{i,j,k}-m_{i,j}{)}^{2}}$ is the standard deviation. Without these standardizations, the largest variable element in the motion data is estimated to be related to performance. To fairly compare all elements in the motion data, the standardization procedure is indispensable.

After standardization, the relevance of motion to performance $\overset{˜}{W}$ is estimated by minimizing the cost function as follows:

$$E(\overset{˜}{W})=\frac{1}{2}\sum_{k}(d_{k}-\sum_{i}\sum_{j}{\overset{˜}{W}}_{i,j}x_{i,j,k}{)}^{2}+\frac{\sigma^{2}}{2}\sum_{i}\sum_{j}{\overset{˜}{W}}_{i,j}^{2}.$$ (7)

The first term on the right-hand side in Eq 7 denotes the minimization of fitting error from motion to performance data. The second term denotes regularization of the norm of $\overset{˜}{W}$ with regularization parameter *σ*^2^. Equivalently, under the existence of independent and identically distributed Gaussian observation noise *ξ*_*i*,*j*,*k*_ associated with *x*_*i*,*j*,*k*_, with a mean of 0 and variance of $\frac{\sigma^{2}}{K}$ (K denotes the total number of trials), minimization of the fitting error with noise averaging $\left\langle E(\overset{˜}{W})\right\rangle$ can be written as follows:

$$\langle E(\overset{˜}{W})\rangle =\frac{1}{2}\sum_{k}\int p(\xi_{1,1,k})d\xi_{1,1,k}...\int p(\xi_{I,J,k})d\xi_{I,J,k}(d_{k}-\sum_{i}\sum_{j}{\overset{˜}{W}}_{i,j}(x_{i,j,k}+\xi_{i,j,k}){)}^{2}$$

or

$$\langle E(\overset{˜}{W})\rangle =\frac{1}{2}\sum_{k}(d_{k}-\sum_{i}\sum_{j}{\overset{˜}{W}}_{i,j}x_{i,j,k}{)}^{2}+\frac{\sigma^{2}}{2}\sum_{i}\sum_{j}{\overset{˜}{W}}_{i,j}^{2},$$

where *p*(*ξ*_*i*,*j*,*k*_) denotes the probability density function of *ξ*_*i*,*j*,*k*_. Thus, the cost function (Eq 7) corresponds to the minimization of the fitting error in the presence of observation noise. In any experimental setting, observation noise is unavoidable. A ridge regression is an effective method with which to estimate the relevance of motion to performance under the existence of observation noise.

Normalization in singular value decomposition: To extract task-relevant spatiotemporal modules, we need to standardize motion data in a different manner from a ridge regression. To estimate motion-relevant spatiotemporal modules, motion data are standardized such that $\frac{1}{JK}\sum_{j}\sum_{k}X_{i,j,k}=0$ and $\frac{1}{JK}\sum_{j}\sum_{k}X_{i,j,k}^{2}=1$. Thus, the averaged value across both time frames and trials should be 0, and the variability across both time frames and trials should be 1 for all spatial elements *i*. For standardization, the original motion data ${\overset{˜}{X}}_{i,j,k}$ should be standardized as follows:

$$X_{i,j,k}=\frac{{\overset{˜}{X}}_{i,j,k}-m_{i}}{s_{i}},$$ (8)

where $m_{i}=\frac{1}{JK}\sum_{j}\sum_{k}{\overset{˜}{X}}_{i,j,k}$ and $s_{i}=\sqrt{\frac{1}{JK}\sum_{j}\sum_{k}({\overset{˜}{X}}_{i,j,k}-m_{i}{)}^{2}}$. To compare task-relevant and motion-relevant spatiotemporal modules, we should utilize the same standardization procedure. Inserting Eq 6 into Eq 8 yields

$X_{i,j,k}=\frac{s_{i,j}x_{i,j,k}+m_{i,j}-m_{i}}{s_{i}}$ or $x_{i,j,k}=\frac{s_{i}X_{i,j,k}-m_{i,j}+m_{i}}{s_{i,j}}$. By using this relation, the relevance of motion to performance $y_{k}=\sum_{i}\sum_{j}x_{i,j,k}{\overset{˜}{W}}_{i,j}$ can be rewritten as follows:

$$y_{k}=\sum_{i}\sum_{j}X_{i,j,k}W_{i,j}+const.=\langle X_{k},W\rangle +const.,$$ (9)

where $W_{i,j}=\frac{s_{i}}{s_{i,j}}{\overset{˜}{W}}_{i,j}$ and $const.=\sum_{i}\sum_{j}{\overset{˜}{W}}_{i,j}\frac{m_{i}-m_{i,j}}{s_{i,j}}$. Because const. does not include both ***X*** and ***W***, it adds some bias to the predicted performance.

After the calculation of Eq 9, we apply SVD to ***W*** as ***W*** = ∑_*r*_*λ*_*r*_***s***_*r*_***t***_*r*_, where ***s***_*r*_ and ***t***_*r*_ are task-relevant spatial and temporal modules. The relevance of motion to performance can be rewritten as

$$y_{k}=\underset{r}{\sum }\lambda_{r}s_{r}^{T}X_{:,:,k}t_{r}+const..$$

#### Relationship between task-relevant modules and motion-relevant modules

In this subsection, we utilize vectorized notations of ***W*** and ***X***_*k*_, i.e., ***w*** = vec(***W***)∈ℝ^[*IJ*,1]^ and ***x***_*k*_ = vec(***X***_***k***_)∈ℝ^[1,*IJ*]^. Additionally, we define a motion data matrix as ***x*** = (***x***_1_,…,***x***_*K*_)∈ℝ^[*K*,*IJ*]^, i.e., a ridge regression can be written as the minimization of cost function $E(w)=\frac{1}{2}(d-xw{)}^{T}(d-xw)+\frac{\sigma^{2}}{2}w^{T}w$. In these formats, the relevance of motion to performance ***w*** can be rewritten as

$$w=(x^{T}x+\sigma^{2}I{)}^{-1}x^{T}y,$$

where **I** denotes an identity matrix, and *σ*^2^ is the same regularization parameter as in Eq 7.

By using SVD, a vectorized motion dataset ***x***_*k*_∈ℝ^[1,*IJ*]^ can be written as $x_{k}=\overset{R_{st}}{\underset{r=1}{\sum }}\omega_{r}u_{k,r}v_{r}$, where *R*_st_ is the number of modules, *ω*_*r*_≥0 is the relevance of the *r*th module used to reconstruct ***x***_*k*_, *u*_*k*,*r*_ denotes how ***v***_*r*_∈ℝ^[1,*IJ*]^ is related to the *k*th trial, and ***v***_*r*_ is the *r*th motion-relevant spatiotemporal module. Notably, ***v***_*r*_ includes both spatial and temporal information in a mixed manner in contrast to an independent manner such as motion-relevant (Eq 1) and task-relevant modules (Eq 3). Thus, the SVD of ***x*** is ***x*** = ***UHV***^T^, where ***U***∈ℝ^[*K*,*K*]^ is an orthonormal matrix, ***H***∈ℝ^[*K*,*IJ*]^ includes the square root of eigenvalues of ***xx***^*T*^ in each diagonal term (i.e., *h*_*i*,*i*_ = *ω*_*i*_, where $\omega_{i}^{2}$ is the *i*th eigenvalue of ***xx***^*T*^), the other terms equal 0, and $V=(v_{1}^{T},\ldots ,v_{\left(I\times J\right)}^{T})\in R^{\left[IJ,IJ\right]}$ is an orthonormal matrix whose row ($v_{i}^{T}$) corresponds to the motion-relevant spatiotemporal module in Eq 5. Notably, this module includes both spatial and temporal modules in an intermixed manner, such as time-varying synergy [44].

Based on the SVD, the relevance of motion to performance ***w*** can be rewritten as

$$w=(x^{T}x+\sigma^{2}I{)}^{-1}x^{T}d=V(HH^{T}+\sigma^{2}I{)}^{-1}V^{T}x^{T}d,$$

where we utilize ***V***^−1^ = ***V***^*T*^ because of its property as an orthonormal matrix. Under the standardization for a ridge regression, ***x***^*T*^ ***d*** = ***β***, where $\beta_{i}=\sum_{k=1}^{K}x_{ki}d_{k}=Cor(x_{i},d)$ = *c*_*i*_. Because the (*r*, *r*)th element of the diagonal matrix (***HH***^*T*^+*σ*^2^**I**)^−1^ equals $\frac{1}{\omega_{r}^{2}+\sigma^{2}}$, the relevance of motion to performance ***w*** satisfies the following:

w=∑r1ωr2+σ2β˜^rvr=∑rf(ωr)gvr(Cor(x,d))vr,

where β˜^r=cvr=gvr(Cor(x,d)), ***c*** = (*c*_1_, *c*_2_,…,*c*_*I×J*_), and $\frac{1}{\omega_{r}^{2}+\sigma^{2}}=f(\omega_{r})$.

#### Motion-relevant modules

The current study utilized SVD to extract motion-relevant spatial and temporal modules. To extract motion-relevant spatial modules invariant across trials, we applied SVD to motion data ***X***_*s*_∈ℝ^[*I*,*JK*]^ as $X_{s}=B_{s}A_{s}C_{s}^{T}$. The motion data were standardized such that $\frac{1}{I}\sum_{i}X_{r,(i,j)}=0$ and $\frac{1}{I}\sum_{i}X_{r,(i,j)}^{2}=1$. The columns of the extracted ***B***_*s*_∈ℝ^[*I*,*I*]^ corresponded to motion-relevant spatial modules invariant across trials. Notably, the extracted modules via SVD are the same as the modules estimated via a PCA.

To extract motion-relevant temporal modules invariant across trials, we applied SVD to motion data ***X***_*t*_∈ℝ^[*J*,*IK*]^. The procedures used to standardize and extract the modules are the same as those used in the motion-relevant spatial modules invariant across trials.

#### Contribution of task-relevant modules to the reconstruction of the original motion data

In Fig 4A, we evaluated the contribution of task-relevant modules to the reconstruction of the original motion data based on a comparison to the contribution of motion-relevant modules. In calculating the contribution of motion-relevant modules (e.g., ***B***_*s*_) to reconstructing the original motion data (e.g., ***X***_*s*_), the eigenvalue matrix $A_{s}A_{s}^{T}$ in $B_{s}X_{s}X_{s}^{T}B_{s}^{T}=A_{s}A_{s}^{T}$ represents the contribution. The *r*th spatial module or the *r*th row of ***B***_*s*_ contributes to the reconstruction of the motion data as $b_{s,r}X_{s}X_{s}^{T}b_{s,r}^{T}=a_{s,r}^{2}$. The normalized contribution to the reconstruction of the motion data by $\hat{r}$ modules was determined as $\sum_{r=1}^{\hat{r}}a_{s,r}^{2}/\sum_{r=1}^{R}a_{s,r}^{2}$, where *R* indicates the rank of the matrix $X_{s}X_{s}^{T}$.

We calculated the contribution of task-relevant modules as follows. First, motion data were set to be ***X***∈ℝ^[*K*,*IJ*]^, where spatial and temporal information was considered together. Second, the relevance of motion to performance was set to be ***w***_*r*_ = vec(***W***_*r*_), where ***W***_*r*_ = *λ*_*r*_***s***_*r*_***t***_*r*_. Third, we calculated ${\hat{\lambda }}_{r}=w_{r}XX^{T}w_{r}^{T}$ in the same manner as that in the motion-relevant modules, although ***w***_*r*_ was not an eigenvector of ***XX***^***T***^. Finally, the normalized contribution to the reconstruction of the motion data by $\hat{r}$ modules was calculated as $\sum_{r=1}^{\hat{r}}{\hat{\lambda }}_{r}/\sum_{r=1}^{R}{\hat{\lambda }}_{r}$.
